# Effectiveness of a Therapeutic Exercise Program to Improve the Symptoms of Peripheral Neuropathy during Chemotherapy: Systematic Review of Randomized Clinical Trials

**DOI:** 10.3390/life13020262

**Published:** 2023-01-18

**Authors:** Snehil Dixit, Valentina Tapia, Carolina Sepúlveda, Daniela Olate, Lily Berríos-Contreras, Luz Alejandra Lorca, Abdulfattah S. Alqahtani, Ivana Leão Ribeiro

**Affiliations:** 1 Department of Medical Rehabilitation Sciences, College of Applied Medical Sciences, King Khalid University, Abha 61471, Saudi Arabia; 2 Department of Kinesiology, Faculty of Healthy Sciences, Universidad Católica del Maule, Talca 3460000, Chile; 3 Hospital del Salvador, Servicio de Salud Metropolitano Oriente, Santiago de Chile 13123, Chile; 4 Department of Rehabilitation Sciences, College of Applied Medical Sciences, King Saud University, Riyadh 11433, Saudi Arabia

**Keywords:** Neoplasia, exercise, sensitivity, chemotherapy, measurement of results reported by the patient

## Abstract

Background: Therapeutic exercise has an important role to manage chemotherapy-induced peripheral neuropathy symptoms. However, there is little evidence of its effectiveness. Objective: To synthesize the evidence regarding therapeutic exercise during chemotherapy to improve peripheral neuropathy symptoms. Databases: PubMed, CINAHL, Cochrane Library, PEDro, ScienceDirect, Scopus, Web of Science and BIREME. Methodology: Randomized clinical trials were included. GRADE was used to synthesize evidence and an inverse variance model for meta-analysis. Results: Up to May 2022, 2172 references were analyzed and 14 studies that evaluated 1094 participants were included. The exercises were highly effective in improving pain threshold and moderately effective in improving peripheral neuropathy symptoms at the 8-week follow-up and the 4–24 weeks. Furthermore, the evidence was low in improving thermal threshold, tactile and vibratory sensitivity. Conclusion: Therapeutic exercise generates a significant reduction in peripheral neuropathy symptoms in patients in short- and long-term follow-up with a moderate level of evidence quality.

## 1. Introduction

Cancer is a global health issue with an increasing incidence and mortality. It is estimated that there will be 18.1 million new cases worldwide and 9.6 million deaths due to this disease [1].

Cancer therapy involves various treatments, such as surgery, chemotherapy, radiation therapy, immunotherapy and hormone therapy [2]. Chemotherapy, being one of the most widely used therapies, uses cytotoxic drugs with the aim of damaging the genetic material of neoplastic cells and preventing their replication [3]. This therapy is not selective; therefore, it damages both tumor cells and healthy cells. As a result of the damage, many side effects are manifested either in the short term such as nausea, neuropathy and fatigue, or in the long term such as premature menopause and cardiac and cognitive dysfunction [4].

Chemotherapy-induced peripheral neuropathy is one of the most debilitating side effects of chemotherapy, since the manifestation of symptoms is linked to the delivered dose of the different chemotherapeutic agents such as platinum compounds, taxanes vinca alkaloids, proteasome inhibitors and epothilones, among others [5].

The prevalence of developing peripheral neuropathy one month after completing chemotherapy is around 68%. The symptoms develop mainly in the hands and feet, with sensory alterations associated with numbness and paresis, motor generating balance and balance problems and autonomous problems with orthostatic hypotension.

Peripheral neuropathy can be evaluated objectively using quantitative sensory tests by performing clinical examinations, as well as subjective measurements such as questionnaires, scales and evaluations of nerve function [6,7]. However, there is no evaluation guideline that is used as a “gold standard”, which generates a great limitation for proper clinical applicability for these patients [5].

Conservative management of chemotherapy-induced peripheral neuropathy involves physical exercise with different types of training, whether aerobic, endurance, motor sensory or balance. Only one systematic review was found, in which it evidenced that a training plan combined with resistance, strength and motor sensory exercises, which should last 36 weeks, at moderate intensities, with a frequency of 2 to 5 days a week and a duration 60 min has been effective in reducing symptoms of chemotherapy-induced peripheral neuropathy [8].

However, studies are lacking that address specific exercise programs for this particular condition [9] and that their results provide clinical applicability. While many studies were found specifying different types of training and subjective measurements of symptoms caused by chemotherapy-induced peripheral neuropathy, many did not identify specific objective and subjective measurements such as the perception of peripheral neuropathy, pressure pain threshold and thermal, tactile and vibration sensitivity.

Considering the above, this systematic review aims to synthesize the evidence regarding interventions with therapeutic exercises during chemotherapy to improve the symptoms produced by peripheral neuropathy with respect to the variables described above.

## 2. Materials and Methods

### 2.1. Study Designation

This systematic review was written according to the preferred reporting model for these study types and meta-analysis (PRISMA) and the recommendations of the Cochrane Collaborations for systematic reviews [10]. The review was registered in PROSPERO with the following number: CRD42020188275. On 9 April 2020, the search began in different databases, regarding various components of the research question considering population, intervention, comparison and results, to identify the knowledge gap; the search was performed until May 2022.

### 2.2. Literature Search

An electronic search of various articles indexed in the following databases was performed: PubMed, CINAHL Plus, Cochrane Central Register of Controlled Trials, Physiotherapy Evidence Database, Science Direct, Scopus and Web of Science y BIREME. The search strategy was adapted for each database. In PubMed, a combination of words was used: (“Neoplasms” [Mesh]) OR cancer AND (exercise) OR “Resistance Training” [Mesh] AND chemotherapy AND (“Peripheral Nervous System Diseases” [Mesh] OR peripheral neuropathy OR pressure pain threshold OR thermal sensitivity OR tactile sensitivity OR vibration sensitivity).

The Start program (version 3.4 BETA, sourced by the Research Laboratory in Software Engineering (LaPES) of the Federal University of São Carlos, Brazil) was used, which was held in the selection of titles, abstracts and full text, considering the analysis between evaluators and consensus criteria. Two independent reviewers (V.T. and D.O.) performed the selection process and two (C.S. and I.L.) participated in the consensus.

### 2.3. Inclusion Criteria

This systematic review included only randomized clinical trials that included therapeutic exercise with a variable related to symptoms of peripheral neuropathy in patients who underwent chemotherapy. The language of the publications was unlimited and should contain a pre-post comparison exercise and that the beginning of the training program was during chemotherapy.

### 2.4. Evaluation of Methodological Quality of the Studies

To Physiotherapy Evidence Database, a PEDro (www.pedro.org.au, accessed on 1 May 2022) scale was used to assess the methodological quality of the studies based on the Delphi list [11]. The studies that were included in this database were previously qualified; if there were no studies, they were manually evaluated by two examiners with a possible consensus by two evaluators. Clinical trials with scores greater than or equal to 6 were considered high methodological, 4 to 5 were rated as moderate quality and lastly, less than or equal to 3 were classified as low methodological quality [12,13].

Data of participants and methodology of the studies were extracted using a standardized form adapted from the Cochrane Collaboration model [10]. Moreover, effect size (ES) with a 95% confidence interval (CI) for continuous outcomes in each comparison group was calculated and the values before and after the intervention were considered. The treatment was classified as small (<0.3), moderate (between 0.4 and 0.7) and large (>0.8) according to Cohen’s index interpretation [14].

The results of the primary studies were interpreted according to the effectiveness of the training programs to improve the symptoms of perception of peripheral neuropathy and increase the pain threshold to pressure, thermal, tactile and vibratory sensitivity. They were considered positive when comparing the intervention and the control groups presented a statistically significant improvement in the primary outcomes.

The preventive effects of therapeutic exercise were rated with an equal sign when there was no difference between the pre and post intervention. Finally, studies that presented a significant reduction in the variables of interest of the intervention were classified as having no effect. The effect size was calculated for studies that presented descriptive ideas, represented by means and standard deviation for the main variables such as pressure, thermal, tactile and vibratory pain threshold.

The synthesis of evidence for each intervention was calculated with the Grading of Recommendations Assessment, Development and Evaluation (GRADE) that considers high, moderate, low or very low evidence level based on the following factors: limitations, indirectness, inconsistency, imprecision and lastly bias risk. In this review, the GRADEpro software (https://gradepro.org, accessed on 1 May 2022) [15] was used to create tables with the synthesis of evidence.

### 2.5. Statistical Analysis

A meta-analysis was performed using means and standard deviations from each selected clinical trial. The difference of standardized means and the 95% confidence interval were calculated using an inverse variance model of random effects for the meta-analysis, considering the data after the intervention. Data heterogeneity between studies were assessed using the I statisitic^2^. The *p* values were calculated and statistical significance was set at <0.05. Statistical analysis was performed using the RevMan five-revision management software (version 5.3, 11-13 Cavendish Square, London, UK).

## 3. Results

**Figure 1:**
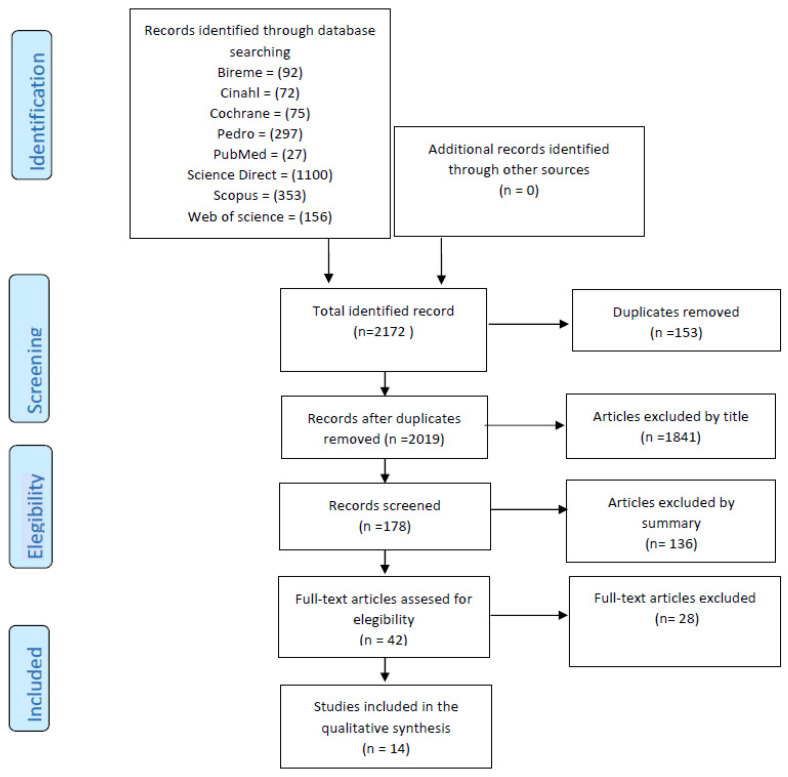
PRISMA flow diagram for the systematic review.

The studies obtained based on the search with the keywords totaled 2.172 articles, which were examined by title and abstracts; after reading the full text of 42 studies, 14 articles were selected that met the inclusion criteria. The flow diagram used is presented in Figure 1.

### 3.1. Evaluation of the Methodological Quality of the Studies

Of the 14 studies collected, 13 were indexed in the PEDro scale [7,16,17,18,19,20,21,22,23,24,25,26]. One [27] was performed manually using the same scale between two evaluators (V.T. and C.S.) in consensus with a third party (D.O.) in case of disagreement. Table 1 shows PEDro scale scores from studies.

Eight of the studies obtained a score equal to or greater than six; therefore, they were classified as having high methodological quality [8,17,18,22,23,26,27,28]. On the other hand, three studies [7,19,20] reached a score between four and five and were categorized as moderate quality. Finally, three studies [16,21,24] obtained a score of three and were therefore cataloged as having low methodological quality. None of the studies scored on the blind allocation criteria for both participants and therapists. In addition, only five studies [17,20,25,27,28] reported that the evaluators who measured at least one key result were blinded. Only six studies [7,19,23,25,26,28] presented results for all subjects who received treatment or were assigned to the control group, or when this could not be, data for at least one key outcome were analyzed by intention to treat (Table 1).

### 3.2. Characteristics of Included Studies

Table 2 describes the main characteristics of the 14 included studies, of which 1094 people participated, having an average age range of 19 to 79 years old, with solid and hematological cancer diagnoses. The comparison was made between a control group, which was based on standard care mainly focused on evaluations and education to patients, and an intervention group, where the main type of exercises that were performed were aerobic training, strengthening of both lower and upper limbs and balance exercises, that began from the first day of chemotherapy treatment or weeks after it. Among the most used equipment in these were the elastic bands, treadmill and pedometer. From 14 studies, five mentioned that intervention was supervised by a physiotherapist [16,17,27,28], one by a nurse [23], one by a sport scientist [21], one by a certified investigator by the ACSM [7], one by an exercise physiologist or oncology nurse [19] and one by a specialist in prescribing exercises for cancer patients [18]. Four studies did not mention the profession of the trainer [20,22,24,26].

The training frequency ranged from 2 to 7 days a week with a duration of 5 to 60 min depending on the exercise performed, a moderate intensity depending on the chemotherapy cycle in which the patients were, and the duration of the protocol varied between 4 and 56 weeks. A physiotherapist supervised 23% of the programs, while the others were supervised by a professional trained to carry out this work. The main variables analyzed were peripheral neuropathy perception, pressure pain threshold and thermal, tactile and vibratory sensitivity. The follow-up was carried out in a period where the minimum range was 4 weeks and the maximum was approximately 36 weeks. The effect size range varied from 0.05 to 0.93 for peripheral neuropathy perception; as for pressure pain threshold, it ranged from 0.44 to 0.57, thermal threshold was 0.06 to 0.28, tactile sensitivity presented a value of 0.01 and vibratory sensitivity presented a value of 0.15.

### 3.3. Synthesis of Evidence

The evidence regarding therapeutic exercises to improve symptoms of peripheral neuropathy, pressure pain threshold, thermal threshold, and tactile and vibratory sensitivity was synthesized according to GRADE with follow-up times of 8 weeks (Table 3) and between 4 weeks and 24 weeks (Table 4). For the synthesis of evidence, two studies were excluded [16,23] because they did not present the necessary data to form part of this analysis, such as control group, intervention, follow-up and the duration of the protocol.

### 3.4. Peripheral Neuropathy Perception

#### 3.4.1. Follow-Up 4 to 24 Weeks

Eight studies [7,17,18,20,21,22,26,27,28] evaluated the peripheral neuropathy perception using questionnaires and scales; with a total of 747 participants in these trials, 52.7% were part of the control group, while 47.2% belonged to the exercise group. According to the factors that can lower the level of quality of the evidence, a score of not serious was presented for risk bias, indirect evidence, imprecision and publication bias, while the inconsistency was categorized as serious since four of the studies were classified as serious and one as very serious, while three of them were not serious. Finally, the therapeutic exercises of strengthening, aerobics, equilibrium and balance presented moderate evidence to improve the symptoms of peripheral neuropathy during chemotherapy for cancer treatment.

#### 3.4.2. Follow-Up at 8 Weeks

Seven studies [7,18,21,22,26,27] evaluated the peripheral neuropathy perception through scales and questionnaires, which included a total of 538 participants of which 53.3% were from the control group and 46.6% from the exercise group. According to factors that may lower the level of quality of the evidence, risk of bias, imprecision and publication bias were categorized as non-serious.

The inconsistency was serious since three studies were classified as serious, one study was classified as very serious and two studies as not serious. Eight weeks of therapeutic exercises to strengthen the upper and lower limbs, equilibrium, aerobics and balance, presented moderate evidence to improve the symptoms of peripheral neuropathy during chemotherapy for cancer treatment.

### 3.5. Pressure Pain Threshold

Two studies [17,19] with a follow-up range of 16 to 18 weeks in which pressure pain threshold was evaluated with an algometer had a total of 254 participants, where 33.8% belonged to the control group and 66.1% to the group with exercises. None of the factors that may lower the level of quality of the evidence were considered serious or very serious. A 16–18-week follow-up with therapeutic nerve gliding, stretching, aerobic and interval resistance exercises presented high evidence for increasing the pressure pain threshold in the trapezium, quadriceps and gluteal areas.

### 3.6. Thermal Threshold

Two studies [7,20] with a follow-up that ranged from 6 to 19 weeks and where thermal threshold was evaluated through questionnaires and scales included 486 participants, of which 51.4% were from the control group and 48.5% from the exercise groups. According to the factors that can lower the level of quality of the evidence, the risk of bias, imprecision, inconsistency and publication bias were categorized as non-serious; however, the indirect evidence was stated as very serious, since both included studies using tools that are not objective for measurement, such as quantitative sensory testing and a numerical scale of 0 to 10.

Along with the above, a follow-up of 6–19 weeks with therapeutic exercises on a vibration platform for the whole body, aerobics and strengthening exercises in the upper and lower limbs presented low evidence to improve the thermal threshold in the lower limb area.

### 3.7. Tactile Sensitivity

Two studies [18,20] with a follow-up range of 12 to 19 weeks evaluated the effects of the therapeutic exercises on tactile sensitivity with questionnaires and Esthesiometer; a total of 158 participants were included, of which 50.6% were from the control and 49.3% were from the exercise group. Regarding the factors that can reduce the level of quality of the evidence, the risk of bias, indirect evidence and publication bias were not serious, while the inconsistency was categorized as serious because two studies were categorized as serious, as well as imprecise because the included studies considered less than 200 participants. Thus, a 12–19-week follow-up with therapeutic exercises to strengthen the lower limbs, aerobics and with a whole-body vibration platform presented low evidence to improve tactile sensitivity in the lower limb area.

### 3.8. Vibratory Sensitivity

Five studies [17,18,22,24,25] with a follow-up range of 4 to 36 weeks evaluated the effectiveness of therapeutic exercises on the vibratory threshold using a quantitative sensitive test and diapason; a total of 183 participants were included, of which 53% were from the control group and 46.9% from the exercise group. According to the factors that can lower the level of quality of the evidence, the risk of bias, indirect evidence and publication bias were not serious, while inconsistency was classified as serious. Only one was not serious, as well as imprecise because the included studies considered less than 200 participants. A follow-up of 4–36 weeks of therapeutic exercises to strengthen the lower limbs, aerobics, balance and motor sensory showed low evidence to increase the vibratory threshold in the lower limb area, such as phalangeal metatarsus, medial malleolus and phalanges, as well as upper limbs such as hands and wrists.

### 3.9. Meta-Analysis

**Figure 2:**
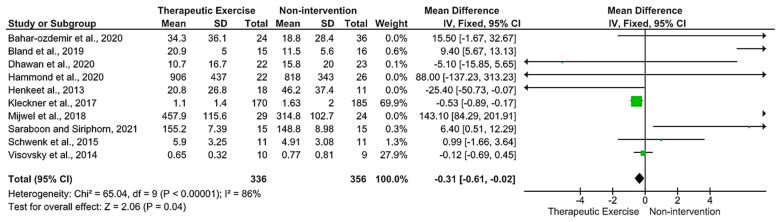
Forest plot of therapeutic exercise program versus control for peripheral neuropathy symptoms [7,16,17,18,19,22,23,26,27,28].

Ten of the fourteen selected studies presented the mean and standard deviation to calculate the effect size (TE) of the intervention [7,16,17,18,19,22,23,26,27,28]. Estimates of the grouped standardized mean difference (DME) showed significant reduction in the symptoms of peripheral neuropathy after a therapeutic exercise program in people with cancer compared to the control group (DME = −0.31; IC 95% = −0.61 to −0.02; *p* = 0.04) (Figure 2), with significant heterogeneity (I^2^ = 86%; *p* ≤ 0.00001).

The sensitivity analysis revealed that heterogeneity was influenced by the studies by Bland et al. 2019 [18] and Mijwel et al. 2019 [19]. There were no changes in the results in favor of therapeutic exercise compared to the control groups and the DME was reduced to −0.40 with changes in heterogeneity from moderate to significant (I^2^ = 45%; *p* = 0.09).

## 4. Discussion

With respect to the quantitative analysis of this systematic review, the findings of the meta-analysis show that a therapeutic exercise program of 4 to 56 weeks generates significant changes, reducing the symptoms of peripheral neuropathy in subjects with cancer compared to the control group with short-term and long-term follow-up. However, given the significant heterogeneity presented, these results should be viewed with caution.

Of the main studies selected, two of them reported improvement of pressure pain threshold after a therapeutic exercise program [17,19]. The study by Mijwel et al., 2018 [13] reported that resistance exercise associated with HIIT significantly improves muscle strength and reduces pain sensitivity; these studies had a moderate effect size with a range of 0.44 to 0.57 and with a high level of evidence.

Regarding the evaluation of neuropathy symptoms through questionnaires, six studies [7,18,21,22,26,27,28] presented moderate evidence with an effect size of 0.27 to 0.47, categorizing them as small to moderate. Three of these studies [7,26,28] reported improvement of peripheral neuropathy symptoms, while two other studies [21,22] kept their symptoms. There was one study [27] that reported an improvement in neuropathic pain symptoms while maintaining generalized pain symptoms after a therapeutic exercise program. The studies that evaluated neuropathic symptoms through thermal threshold [7,20], tactile sensitivity [18,20] and vibratory sensitivity [17,18,22,24,25] presented a low level of evidence.

In regard to the studies that evaluated tactile sensitivity [18,20] and thermal threshold [7,20], no significant differences were obtained. The effect size of the studies [7,20] that evaluated thermal was 0.28 and 0.06, considered small. Likewise, the study [20] that evaluated tactile sensitivity had a small effect size of 0.01. The study that evaluated the vibratory threshold [22] presented a small effect size with a value of 0.15. Those results could be explained by the fact that symptoms of peripheral neuropathy induced by chemotherapy should be assessed by both objective methods and from a patient perspective [29]. Moreover, the heterogeneity of evaluation tools used in those studies negatively impacts these results.

The findings reported in this review are similar to the study by Dobson et al., 2014 [30] that evaluated the effects of balance and aerobic exercise training on neuropathy symptoms, sensory dysfunctions and increased peripheral nerve conduction velocity. However, the review by Dobson et al., 2014 [30] did not determine the quality of evidence and included studies that focus on diabetic neuropathy and its neuro-inflammatory etiology and did not incorporate studies where the neuropathy is the result of exposure to chemotherapy.

The present systematic review determines that strengthening, aerobic and motor sensory exercises for a total average time of 14 weeks (considering a standard deviation of 9 weeks) 2 to 5 days per week and with low to moderate intensities present a moderate level of evidence for the improvement of neuropathy symptoms. These facts coincide with the results of the study by Kneis et al., 2019 [31] that reported that resistance and equilibrium exercises reduced sensitivity symptoms, with an improvement in the physical function of the cancer survivors.

According to the above and considering that there is still controversy regarding the effectiveness of drugs in the management of peripheral neuropathy [32], multimodal exercise emerges as an effective and safe therapeutic tool to reduce peripheral symptoms induced by chemotherapy.

Some limitations of the present study should be considered: (1) three studies showed poor methodological quality [10,15,18]; (2) different therapeutic exercise protocols were used; (3) significant heterogeneity (I^2^ = 86%) which is due to the different types of exercises between studies and different evaluation tools used, providing variability; (4) the selected studies used distant tools to evaluate peripheral neuropathy; (5) two studies [7,20] used methods to evaluate the peripheral neuropathy that were inadequate, since they lacked reliability and objectivity; (6) non-specificity of therapeutic exercise in terms of its dosage; (7) the beginning of the therapeutic exercise program was nonspecific since some studies began together with the chemotherapy [17,27], on the first day of chemotherapy [7,16], before starting it [18,23], during this therapy [24,25] or on subsequent days to the start of chemotherapy [18,19]. In addition, three studies [20,22,26] did not specify the beginning of the intervention protocol; (8) Three studies [20,22,24] did not identify any supervisor, which may limit the correct execution of the exercises; (9) different follow-up times between studies can generate a bias in the results.

## 5. Conclusions

In general, this systematic review and meta-analysis suggest that therapeutic exercise generates a significant reduction in peripheral neuropathy symptoms in patients in short and long-term follow-up with a moderate level of evidence quality. However, these results must be viewed with caution due to the significant heterogeneity of the studies analyzed. The available studies are diverse in terms of methodology, exercise dosage, and tools to assess peripheral neuropathy; therefore, further research is warranted. Future clinical trials must present adequate methodological quality and use valid and reliable evaluation methods.

## Figures and Tables

**Table 1:** Identification of the 14 studies included in the review classified according to the PEDro scale.

Author	1	2	3	4	5	6	7	8	9	10	11	Total
Henke, et al., 2014 [16]	1	1	-	-	-	-	-	-	-	1	1	3
Bahar-Ozdemir Y et al., 2020 [27]	1	-	1	1	-	-	1	1	-	1	1	6
Hammond E et al., 2020 [17]	1	1	1	1	-	-	1	-	-	1	1	6
Bland, K. A et al., 2019 [18]	1	1	1	1	-	-	-	1	-	1	1	6
Mijwel et al., 2018 [19]	-	1	-	1	-	-	-	-	1	1	1	4
Schönsteiner et al., 2017 [20]	-	1	-	1	-	-	1	1	-	1	1	5
Kleckner IR et al., 2017 [7]	-	1	1	1	-	-	-	-	1	1	1	5
Vollmers, P et al., 2018 [21]	1	1	-	-	-	-	-	-	-	1	1	3
Schwenk et al., 2016 [22]	1	1	1	1	-	-	-	1	-	1	1	6
Visovsky et al., 2014 [23]	1	1	1	1	-	-	-	-	1	1	1	6
Stuecher, K. et al., 2018 [24]	1	1	-	-	-	-	-	-	-	1	1	3
Streckmann F et al., 2014 [25]	-	1	-	1	-	-	1	1	1	1	1	6
Dhawan S et al., 2020 [26]	1	1	1	1	-	-	-	1	1	1	1	7
Saraboon, C et al., 2021 [28]	1	1	1	1	-	-	1	1	1	1	1	8
	10/14	13/14	8/14	11/14	0/14	0/14	5/14	7/14	6/14	14/14		

1. Were the eligibility criteria specified? 2. Were the participants randomly allocated between the groups? 3. Was the allocation blinded? 4. Were the groups similar at the baseline for the most important prognostic indicators? 5. Were the participants blinded? 6. Were the therapists who performed the intervention blinded? 7. Were the evaluators who measured at least one measure of response blinded? 8. Did the measures of at least one outcome affect >85% of the participants initially allocated to the groups? 9. Did all the participants receive the treatment or a control condition; if not, were the data analyzed with intention-to-treat analysis? 10. Did the statistical comparison results between groups report at least one key response variable? 11. Did the study present reliability measures for at least one variable response?

**Table 2:** Characteristics of the studies included in the review.

Study	Participants, Mean ± DE of Age	Start of Intervention	Comparison Groups and Training Type	Frequency and Duration of Training/Protocol	Outcomes	Follow-Up	Main Results/Magnitude of Effect
Henke, et al., 2014 [16]	29 Participants IG: 18 CG: 11 IG: NI CG: NI	First day of Che	IG: Aerobic and whole body strength training CG: Conventional physical therapy (breathing techniques and manual therapy)	Aerobic: 6 min a day/5 times per week Strengthening: 2 times per week, 10 repetitions per exercise at their maximum capacity/NF	Quality of life (Peripheral neuropathy) Questionnaire	NF	Peripheral neuropathy (+)/ES PI = −0.81 (−1.52; 0.02); ES IG = −0.64 (−1.25; 0.08); ES CG = −0.41 (−1.22; 0.46)
Bahar-Ozdemir et al., 2020 [27]	60 Participants IG: 24 CG: 36 IG: 52 ± 9.99 years CG: 53.58 ± 11.92 years	Started together with Che	IG: Strengthening with LL resistance exercises and equilibrium/balance exercises. CG: Guidance regarding PA	Strengthening: 2 sets/10 rep. 5 times per week Equilibrium: 10 min of exercise/5 days a week/10 weeks	Neuropathic pain Questionnaire	Post. Third round of Che	Neuropathic pain (+), IG: 26.3%, CG: 47.2% Pain (=)/ES PI = 0.47 (−0.09; 0.95)
Hammond E et al., 2020 [17]	48 Participants IG1: 22 CG: 26 IG2: 56.3 ± 9.9 years CG: 53.0 ± 10.3 years	Started together with Che	IG: Nerve gliding exercises, stretching, ROM. and education CG: Standard care and nerve reevaluations	5 to 10 min./3 times a day/24 weeks	Pain report, neuropathic pain, vibratory threshold, pressure pain threshold. Numerical scale, survey, The TSAII Vibration Sensory Analyzer, algomeESr	Baseline, PChe, 12 weeks and 24 weeks	Pain (+), no pain report, IG: 70.1%; CG: 51% Neuropathic pain (=)/ES = NI Vibratory threshold (=)/ES = NI Pain pressure threshold (+)/ES PI= 0.54 (−0.08; 1.07) ES PI 3 months = 0.65 (0.01; 1.18); ES PI 6 months = 0.23 (−0.37; 0.79)
Bland, K. A et al., 2019 [18]	27 Participants IG1:12, CG:15 IG2: 51.0 ± 8.1 CG: 49.5 ± 11	IG: 1 week before Che. CG: 2 to 3 weeks post Che	Aerobic and LL strengthening exercises in both groups	Aerobic: 5 days/week, 50% to 75% HRR and Borg of 12 to 14 in a classification of 6 to 20, 15 to 30 min, progressive. Strengthening: 1 to 2 sets of 10 rep. at 50% to 65% 1RM, progressive/10 weeks	CIPN, Vibratory threshold, Tactile threshold. Questionnaire, Diapason, Esthesiometer	10 to 15 weeks	CIPN, sensory symptoms PI (=)/ES = −0.11 (−0.86; 0.66); (=) follow-up, ES = 0.34 (−0.45; 1.07)/CIPN, motor symptoms PI (=)/ES = 0.14 (−0.63; 0.89); (=) follow-up, ES < 0.01 (−0.76; 0.76)/CIPN, autonomic symptoms PI (=)/ES = 0.30 (−0.48; 1.04); (=) follow-up, ES = 0.54 (−0.27; 1.27)/ Vibratory threshold PI (=)/IG, 59% with vibratory symptoms; CG, 68% with vibratory symptoms/Tactile sensitivity (=)/NI
Mijwel et al., 2018 [19]	206 Participants IG1: 74 IG2: 72 CG:60 IG1: 52.7 ± 10.3 years IG2: 54.4 ± 10.3 years CG: 52.6 ± 10.2 years	3 days after second Che Session	IG1: conventional resistance exercises with high intensity intervals IG2: Continuous aerobic exercises of moderate intensity CG: Standard care	IG1: 2 days per week, 2 or 3 sets of 8 to 12 rep. at an intensity of 80% of 1RM. IG2: 20 min of continuous aerobic exercise, 2 days per week IG1 y IG2: 3 × 3 min HIIT with an of RPE 16–18 interspersed with 1 min recovery/16 weeks	Pain pressure threshold. Algometer	Baseline and 16 weeks	Trapezium PPT, taxanes (=)/ES IG1xCG = 0.27 (−0.20; 0.71); ES IG2xCG = −0.16 (−0.59; 0.29) Gluteal PPT, taxanes (=)/ES IG1xCG = 0.14 (−0.32; 0.58); ES IG2xCG = −0.12 (−0.56; 0.34) Trapezium PPT, without taxanes (+)/ES IG1xCG = 1.30 (0.61; 1.79)/ES IG2xCG = 0.66 (0.04; 1.19) Gluteal PPT, without taxanes (+)/ES IG1xCG = 1.03 (0.38; 1.52)/ES IG2xCG = 0.82 (0.18; 1.34)
Schönsteiner et al., 2017. [20]	131 Participants IG: 66 CG: 65 IG: 59 (range: 28–70) years CG: 62 (range: 24–71) years	NI	IG: Training with whole body vibration platform. CG: Posture and transport movements training. IG + CG: Massages and passive mobilization	15 sessions, 2 times/week, with warm-up of 3 min per session, 9–23 Hz with progressive increments of 12 min with progression of 9–13 Hz during 9 min. Massage and passive mobilization for 30 min./15 weeks	Peripheral neuropathy, Quantitative evaluation of paresthesia, Thermal and Tactile threshold. Questionnaire Diapason. Quantitative sensory tests	Baseline, 4 weeks, 8 weeks post last intervention	Peripheral neuropathy symptoms in LL PI (+)/reduction of numbness from 97 to 81% and discomfort from 98 to 71%; Thermal threshold to hot PI (=)/ES = −0.15 (−0.56; 0.28); Heat pain threshold PI (=)/ES = −0.01 (−0.43; 0.41); Thermal threshold to cold PI (=)/ES = 0.02 (−0.40; 0.44); Tactile sensitivity (=)/ES = 0.01 (−0.41; 0.43)
Kleckner IR et al., 2017 [7]	355 Participants IG: 170 CG: 185 IG: 55.6 ± 11.8 years CG: 55.9 ± 9.7 years	First day of Che	IG: Standard care and exercise (aerobic and strengthening of UL and LL) CG: Standard care (completed all assessments and intervention at the end of the study)	60 min/week Aerobic: 60–85% HRR, progressing 5–20% each week. Strengthening: Low to moderate intensity, dependent on elastic bands, RPE valued at 3 to 5./6 weeks	Peripheral neuropathy, numbness and tingling, hot/cold. Scales	Baseline and post 6 weeks	Peripheral neuropathy, numbness and tingling (+)/ ES PI = −0.29 (−0.47; −0.06); hot and cold in extremities (+)/ES PI = −0.28 (−0.47; −0.05)
Vollmers P et al., 2018 [21]	36 participants IG: 17 CG: 19 IG:48.56 ± 11.94 years CG: 52.39 ± 10.14 years	At the start of Che	IG: Regular physical training and motor sensitive exercises. CG: Brochure with information and suggestion of PA	Intensity depends on the physical state of the participant/56 weeks	Neuropathic symptoms. Questionnaire	Baseline and PAter 6 weeks post Che	Neuropathic symptoms. (=)/NI
Schwenk et al., 2016 [22]	19 Participants IG: 9 CG: 10 IG: 68.73 ± 8.72 years CG: 71.82 ± 8.85 years	NF	IG: Equilibrium and balance exercises CG: Encouraged to remain active	2 45 min. sessions/week for 4 weeks./4 weeks	Pain, Vibratory threshold: Numbness in feet. Scales	Ev. Baseline and after 4 weeks	Pain (=)/ES PI = 0.31 (−0.55; 1.13); Vibratory threshold (=)/ES PI = −0.15 (−0.98; 0.69); Numbness in feet (=)/ES PI = 0.31 (−0.55; 1.13)
Visovsky et al., 2014 [23]	19 Participants IG: NE CG: NE 48.8 (range 24–65) years	Before starting Che	IG: Aerobic and resistance exercises for LL and UL. CG: ACS standardized brochures	5–7 days for 20 min in intervals with a light to moderate intensity Strengthening: 3 times per week, 1–3 sets of 8–12 progressive strength exercises./12 weeks	Peripheral neuropathy. Questionnaire	Baseline, 4, 8, 12 and 24 weeks	Peripheral neuropathy (=)/ES PI 4 weeks = 0.96 (−0.06; 1.83); ES PI 8 weeks = 0.14 (−0.77; 1.03); ES PI 12 weeks = 0.34 (−0.60; 1.22); ES PI 24 weeks = 0.73 (−0.25; 1.60)
Stuecher, K. et al., 2018 [24]	28 Participants IG: 13 CG: 15 IG: 66.8 ± 7.8 years CG: 65.9 ± 7.9 years	During Che	IG: Aerobic training. CG: Standard care based on hospital oncologist guidelines	IG: moderate intensity, RPE + Borg, classification of 11–13 on the 6–20 scale, progressive until reaching 150 min per week./12 weeks	Vibratory threshold. Diapason	From 4 to 6 weeks and after week 12	Peripheral neuropathy (=)/NI
Streckmann F et al., 2014 [25]	61 Participants IG: 30 CG: 31 IG: 44 (range: 20–67) years CG: 48 (range: 19–73) years	In the first round of chemotherapy	IG: Standard care and training (aerobic, motor sensory and strengthening) CG: Standard routine care	Frequency: 2 times per week Aerobic: Start: (60%−70% of HRM) Final: 10 to 30 min. (70%–80% of HRM) Motor sensory: postural stabilization, progressive, in 3 sets/20 s. between each set and 1 min between exercise. Strengthening: 4 exercises during 1 min with maximum force./36 weeks	Vibratory threshold. Diapason	Baseline, 12, 24 and 36 week follow-ups	Vibratory threshold PI 36 weeks (+)/IG reduced 87.5% of the symptoms compared to CG (0%)
Dhawan S et al., 2020 [26]	45 participants GE: 19 CG: 22 GE: 50.5 ± 7.9 years CG: 52.5 ± 6.6 years	NF	GE: Muscle strengthening and balance exercises. CG: Standard routine care	30 min. a day/convenience./10 weeks	Neuropathic pain. Peripheral neuropathy symptoms experience. Questionnaire	Baseline, 10 week follow-up	Neuropathic pain (+)/ES PI = −0.28 (−0.85; 0.33); Peripheral neuropathy symptoms experience (+)/ES PI = −0.37 (−0.93; 0.25)
Saraboon C et al., 2021 [28]	30 participants GE: 45.07 ± 3.88 years GC: 45.53 ± 4.64 years	Before starting Che	GE: Balance, aerobic and stretching exercises, plus 10 min rest between each exercise. GC: conventional therapy plus balance exercise program if desired	Frequency: 2 times per week for 6 weeks balance 10 rep at 40 min, aerobic 5 min of cycling, 5 min of stretching once a day./6 weeks	Symptoms of peripheral neuropathy (Michigan Diabetic Neuropathy Score: MDNS) Quality of life (FACT scale -Taxane)	Baseline, 4 and 6 week follow-up	Symptoms of peripheral neuropathy (+)/0.30 (−0.44; 0.99) Quality of life (=)/0.19 (−0.54; 0.89)

±: Standard deviation; PI: Post intervention; Che: Chemotherapy; CIPN: Chemotherapy-induced peripheral neuropathy; IG: Intervention group; CG: Control group; NF: Not found; Post.: Posterior; HRM: Maximum heart rate; min: Minutes; s: Seconds; rep.: Repetitions; ROM: Range of motion; LL: Lower limbs; UL: Upper limbs; RM: Maximum repetition; RPE: Scale of perceived exertion; ACS: American cancer society; Ev.: Evaluation; PPT: Pressure pain threshold; HIIT: *High Intensity Interval Training*; PA: Physical activity; (+): Positive effect of the treatment; (=): Treatment with no effect; ES: Effect size.

**Table 3:** Summary of the evidence for the perception of peripheral neuropathy with an 8-week follow-up according to GRADE.

Certainty Assessment							Summary of the Results			
							Study Event Rates (%)		Anticipated Absolute Effects	
Participants (Studies) Follow-Up	Risk of Bias	Inconsistency	Indirect Evidence	Imprecision	Risk of Publication	Overall Certainty of Evidence	Control Group	Exercise Group	Control Group Risk	The Risk Difference with Exercises
**Peripheral Neuropathy Symptoms** (**Evaluated with: Questionnaire and Scale**)										
538 (6 Random trials)	Not serious	Serious ^a^	Not serious	Not serious	Neither	**⨁⨁⨁◯** **MODERATE**	287/538 (53.34%)	251/538 (46.65%)	The mean symptoms of peripheral neuropathy Follow-up 2 months was 0	Mean 0.33 (Range: 0.7 to 0.7)

GRADE: Grading of Recommendations Assessment, Development and Evaluation. Event rates: number of patients in the intervention or comparison group/total study participants (% of patients in each intervention or comparison group used according to the GRADE recommendation). The term in bold refers to the level of evidence according to GRADE. ^a^: <75% of the studies report that the intervention presented positive or negative results.

**Table 4:** Summary of the evidence for the variables of perception of peripheral neuropathy, pressure pain threshold, vibratory pain, tactile and thermal sensitivity in a follow-up of 4 to 24 weeks according to GRADE.

Certainty Assessment							Summary of the Results			
							Study Event Rates (%)		Anticipated Absolute Effects	
Participants (Studies) Follow-Up	Risk of Bias	Inconsistency	Indirect Evidence	Imprecision	Risk of Publication	Overall Certainty of Evidence	Control Group	Exercise Group	Control Group Risk	The Risk Difference with Exercises
**Peripheral Neuropathy Symptoms** (**Assessed with: Questionnaires and Scales**)										
747 (9 Random trials)	Not serious	Serious ^a^	Not serious	Not serious	Neither	**⨁⨁⨁◯** **MODERATE**	393/747 (52.71%)	354/747 (47.28%)	The mean peripheral neuropathy symptoms was **0**	Mean 0.33 (Range: 0.93 to 1.13)
**Pressure Pain Threshold** (**Evaluated with: Algometer**)										
254 (2 Random trials)	Not serious	Not serious	Not serious	Not serious	Neither	**⨁⨁⨁⨁** **HIGH**	86/254 (33.85%)	168/254 (66.14%)	The mean pressure pain threshold was **0**	Mean 0.37 (Range: 0.59 to 1.34)
**Vibratory Threshold** (**Evaluated with: Sensitive Quantitative Test and Tuning Fork**)										
183 (5 Random trials)	Not serious	Serious ^b^	Not serious	Serious ^c^	Neither	**⨁⨁◯◯** **LOW**	97/183 (53%)	86/183 (46.99%)	The mean vibratory threshold was **0**	Mean 0.15 (0.98 to 0.69)
**Tactile Sensitivity** (**Evaluated with: Questionnaire and Esthesiometer**)										
158 (2 Random trials)	Not serious	Serious ^d^	Not serious	Serious ^e^	Neither	**⨁⨁◯◯** **LOW**	80/158 (50.63%)	78/158 (49.36%)	The mean tactile sensitivity was **0**	Mean 0.01 (Range: 0.41 to 0.43)
**Thermal Sensitivity** (**Evaluated with: Questionnaire and Scale**)										
486 (2 Random trials)	Not serious	Not serious	Very serious ^f^	Not serious	Neither	**⨁⨁◯◯** **LOW**	250/486 (51.44%)	236/486 (48.55%)	The mean thermal threshold was **0**	Mean 0.17 (Range: 0.56 to 0.44)

GRADE: *Grading of Recommendations Assessment, Development and Evaluation*. Event rates: number of patients in the intervention or comparison group/total study participants (% of patients in each intervention or comparison group used according to the GRADE recommendation). The term in bold refers to the level of evidence according to GRADE. ^a,b,d^: <75% of the studies report that the intervention presented positive or negative results. ^f^: Heterogeneity in relation to the intervention protocols used. ^c,e^: Results based on a total sample of <200 participants.

## Data Availability

All data are from the primary included studies in this systematic review and are referenced in the article.
